# Miscellaneous Notices

**Published:** 1855-10

**Authors:** 


### MISCELLANEOUS NOTICES.

*Supposed Injustice*—We had no intention of doing injustice to any one in the note referred to in the following communication, but knowing, as we did, that a description of the *"compound screw forceps"* had been given to the profession by Dr. S. P. Hullihen through the pages of the Journal, before Dr. Dub's ratchet-screw instrument was generally known, and being under the impression that the ratchet was all that was patented, we thought it right to state the fact, and in so doing, "render unto Cæsar the things which are Cæsar's."—Eds.

Natchez, *September 4th*, 1855.

Messrs. Editors :—In the July number of your Journal, you published a letter from Dr. C. H. Dubs, making over to the profession his "union screw forceps," to which you appended some remarks tending to ignore his claims to the invention, and at the same time alleging, that the only difference between his instrument and Dr. Hullihen's is, "that the screw of Dr. H's is moved by means of a spiral spring and Dr. D's by a ratchet." Although this in itself constitutes a wide difference between the two instruments, a fact well known to all who have used both, it is not the only one, but as it is not my intention to trespass on your time by entering into a detailed examination of the merits of either instrument, I will merely remark, that the comments I have alluded to above, are, to say the least of them, uncourteous towards Dr. Dubs, and not at all calculated to serve the profession; for it is a simple truth, that the more encouragement any profession gives to the exercise of talent and genius among its members, the more extended will be its research, and the more rapid its progress towards perfection, no matter how small the contribution may be either in advancing its science or improving its appliances. Hoping that in your forthcoming number you will "render unto Cæsar the things which are Cæsar's," I remain, gentlemen, your ob't serv't,

W. A. MARTIN.

---

*Premature Dentition.*—Dr. Smull, of Baltimore, presented to the senior editor a few weeks since, the cap of an upper temporary central incisor, which made its appearance through the gums four days after the birth of the child. Four days after it dropped out. The process of dentinification had only proceeded just far enough to form a thin shell over the pulp, which is but very imperfectly covered with enamel. A few examples are on record of children being born with two or more teeth through the gums—one such has come under the observation of the writer. Such examples, however, are very rare.

The child in this case was born at its full time, but has had convulsions since its birth, and is now, as we are informed by Dr. Smull, affected with marasmus.

*Porcelain Teeth.*—Dr. McCurdy, of the firm of Jones, White & McCurdy, has presented to the senior editor some very beautiful specimens of porcelain teeth, made from new moulds recently gotten up by these enterprising manufacturers. The specimens consist of gum teeth without the gum color, designed to be mounted on a platina base—the gum-enamel to be afterwards applied and fused on in a furnace; thus making it continuous around the entire arch, without seam or break, and uniting the teeth to each other. They are said to be an improvement on Allen & Hunter's method of mounting, but whether they possess any real advantages over it or not, we are unprepared to say—not having had an opportunity as yet of putting them to a practical test. There are also several single gum teeth and several blocks of twos, and a few designed to be mounted with continuous gums. The entire collection presented to the editor are very beautiful, having, in an eminent degree, the peculiar life-like dentinal translucency so necessary, but so seldom seen, in porcelain teeth. It is a point at which all manufacturers should aim.

*American Society of Dental Surgeons.*—We publish in another part of the present number of the Journal, the proceedings of a meeting of the above named society, held 8th May, 1855, at Cincinnati, O. This meeting adjourned to the 1st of August, to meet in Philadelphia, for the purpose of discussing, among other topics, the expediency of dissolving the Association. The subject, at the last meeting, elicited remarks from several gentlemen, and was finally referred to a committee. This committee reported after some deliberation, that it was not deemed expedient to dissolve the society at present. The same committee were continued, with instructions to amend the constitution and by-laws, if they thought the advancement of the society required it, so as to diminish the initiation fee and annual dues, and do such other things as may be necessary to place the society upon a more permanent basis. The society then adjourned to meet the first Tuesday of August, 1856, at the Astor House in the city of New York.

*Amalgam.*—We regret to learn that this compound is becoming more extensively employed than ever, for filling the cavities of decayed teeth. It is true that it is a cheaper material and may be packed into a cavity far more expeditiously than gold or tin foil. This makes it a desirable filling for the operator. The patient, however, here, as in many other instances, has to suffer the dentist's convenience. Mercury is an undoubted poison, a poison of slow, steady, but certain malignity; a poison, even in its insoluble combinations, capable of producing grave and lasting disturbances of health.

The only question to be determined then is, whether the combination of this injurious metal with other substances, diminishes or prevents its pernicious action upon the living body. We are firmly convinced that it does not, that mercury in the amalgam introduced into cavities of teeth, is still mercury, capable of solution in the fluids of the mouth and absorption by the proper vessels. To the demonstration of this proposition we propose to devote an article in the January number of the Journal. We think we can prove satisfactorily to all unbiased minds, the dangerous character of this compound.

*Skillful Extraction of Teeth.*—We had thought that in these days of scientific dentistry the old bungling method of tearing and wrenching the gums and teeth from the maxilla was a thing almost unknown, until a few weeks since, a young man called upon us with the whole of the alveoli, containing the three molars, and one bicuspid torn from the left upper jaw, presenting it as a testimony of the "things that are;" a prosecution had been entered against the butcher, under claim of damage to the amount of $200, which of course has been most readily accepted and paid.

And this done in one of the largest cities of the United States. Is it not time that dental education should be peremptorily required by all communities? A. A. B.

*A Lizard removed from the throat of a child.*—A little girl about five years of age was brought to the senior editor a few days since to have a tooth extracted; after the operation was performed, an uncle of the child who accompanied it informed him, that some two years before she had swallowed, while drinking in the dark, a lizard, which had remained in her stomach nearly twenty months; during which time her health was so much affected, that her life, for some months, was despaired of. But at the expiration of the time above mentioned, she felt something in her throat, and putting her hand in her mouth, pulled out a live lizard, more than three inches in length. Her health immediately began to improve, and in a short time was completely restored.

*Tooth Soap.*—We have received from Mr. E. McClain, of Philadelphia, a sample of a detergent soap for the teeth, a substitute for dentifrice. He calls it Peruvian tooth soap, and tells us it is composed of Peruvian bark, myrrh, orris root, bole armeniæ, and the best olive oil soap. We have used it and found it agreeable and effective.

*A Brace of Worthies.*—It has often been remarked, that two of a trade seldom agree. In the following report of a trial of an action for damages, for injuries received in extracting a tooth, this erudite proverb is exemplified. It must be understood, the parties referred to are what is termed mere tooth drawers; we particularly direct attention to the evidence of the plaintiff's witness, a Mr. Hayes, whose ***graphic and learned description*** of the injuries received by the plaintiff, and the ***mortification*** that had commenced and was going on, at the time his professional services were required. According to his evidence, it appears he considered it necessary to extract two sound teeth to enable him to get at the *mortified* fang. No doubt local injury had been done in the attempt to extract the tooth, and the patient suffered in consequence, and Mr. Hayes unquestionably thought it a famous opportunity to obtain a temporary popularity and importance; he would see his name in print, and did not hesitate in giving a humane dig at his adversary "Morgan;" for what we know, he might have suggested the action for damages. It is quite evident, the medical men first consulted, were not quite easy in the company they were likely to be in, and, therefore, did not appear to substantiate the *mortified* evidence of Mr. Hayes. It would have been highly interesting to have heard this evidence rebutted by the defendant, Morgan.

The following is the case as reported in the English papers:

BAKER VS. MORGAN AND ANOTHER.

Mr. Edward James and Mr. Milward were for the plaintiff; Mr. Edwin James and Mr. Hawkins for the defendants.

The plaintiff is a porter, and brought the present action to recover from the defendants, who are dentists, in Finsbury-place, compensation for an injury done to him in extracting a tooth. It appeared that in the month of February last, the plaintiff, being a martyr to the tooth-ache, went to the house of the defendants to have his tooth drawn. The operation was attempted to be performed, (the precise person who operated, however, being uncertain,) but in the course of it, the tooth, which was very much decayed, broke, and the fangs were left in the jaw. These the plaintff desired to have extracted, but the operator thought that it would be better to let them remain for the present, and requested Mr. Baker, if he felt any pain, to call again in a day or two. Two or three days elapsed, and the pain not having subsided, the plaintiff, instead of going back to Messrs. Morgan, consulted Dr. Epps, of Great Russell street, who sent him to Mr. Hayes, a dentist on Conduit street. This gentleman was called, and described the plaintiff's suffering as intense. His face was so swollen that his eyes were almost closed, and he thought it necessary to request the assistance of and advice of another medical man, Dr. M'Oubrey. Mortification had commenced, an abominable stench proceeded from the mouth, splinters were taken from the jaw, which was broken, two large holes were found in the upper part of the palate, arising, according to his opinion, from unnecessary violence and an improper use of the "key," instead of the forceps, and two of the firm teeth were necessarily extracted in order to get at the broken fangs. Neither Dr. Epps, however, nor Dr. M'Oubrey was called, and it was not till after the lapse of some weeks that any intimation of the plaintiff's injury was given to the defendants.

On the other side, neither of the defendants nor their assistants had any recollection of the plaintiff; one of them might have extracted his tooth, but they were all quite confident that they had never broken the jaw of any one; in fact, in this case it would have been impossible, since the tooth, being greatly decayed, broke off with but a very slight wrench. Several professional men testified to their skill as dentists, and it having been suggested that the injuries, if not exaggerated by Mr. Hayes, might now be easily discernible.

Mr. Gay. accompanied by Mr. Hayes, retired to examine the state of the plaintiff's jaw, and on their return, concurrred in the opinion that no material injury now existed.

The Chief Baron then summed up, and the jury immediately returned a verdict for the defendants.

